# A call to action—stopping antimicrobial resistance

**DOI:** 10.1093/jacamr/dlac142

**Published:** 2023-01-07

**Authors:** Robert W Eisinger, Mark P Williams, Sung Hee Choe, Esther Krofah

**Affiliations:** FasterCures, Milken Institute, 730 15th Street, Washington, DC 20005, USA; FasterCures, Milken Institute, 730 15th Street, Washington, DC 20005, USA; FasterCures, Milken Institute, 730 15th Street, Washington, DC 20005, USA; FasterCures and Center for Public Health, Milken Institute, 730 15th Street, Washington, DC 20005, USA

## Abstract

Antimicrobial resistance is a public health emergency and represents an impending pandemic. Implementing the lessons learned from responding to the COVID-19 pandemic is essential in accelerating the development of new antimicrobials and therapeutic strategies, rapid diagnostics and improved vaccines. A rededicated, coordinated and collaborative global effort of all stakeholders in academia, healthcare, government agencies, industry, finance and philanthropy is essential to halt the continued spread of antimicrobial resistance and to prevent further morbidity and mortality from drug-resistant pathogens.

Antimicrobial resistance (AMR) represents an ongoing public health emergency and an impending pandemic of overwhelming proportions. The WHO has declared AMR among the 10 worldwide public health threats facing humanity.^1^ In 2019, it is estimated that 4.95 million deaths globally were associated with bacterial antibiotic resistance. This staggering statistic included 1.27 million deaths directly attributed to bacterial AMR. These estimates suggest that bacterial AMR caused more deaths in that year than those resulting from HIV/AIDS and malaria combined. The major burden of AMR deaths is reported in low-resource areas—those with limited healthcare systems, laboratory infrastructure, sanitation, clean water and antibiotic stewardship.^2^ In the US, the CDC reports that there are more than 2.8 million antibiotic-resistant infections and greater than 35 000 deaths associated with AMR each year.^3^ A coordinated, global effort is needed to halt the continued spread of AMR and the resulting morbidity and mortality from drug-resistant pathogens. This Call to Action requires increased support for biomedical research, as well as significant expansion of and incentives for new and improved diagnostics, antimicrobials and vaccine candidates in the medical diagnostic and pharmaceutical pipelines, respectively.

During the past 2.5 years, the ongoing COVID-19 pandemic has served to further accelerate the AMR public health crisis with the increased use of antibiotics in the treatment of patients with SARS-CoV-2, the virus that causes COVID-19, as well as resulted in major disruptions in healthcare systems and infectious disease monitoring and treatment.^4^ The COVID-19 pandemic has underscored the critical role of biomedical research and development in responding to public health emergencies with accurate, easy-to-use and rapid medical diagnostics, safe and effective vaccines and therapeutics, as well as public–private partnerships resulting in the essential infrastructure for community engagement in clinical trials and uptake of medical countermeasures.^5^ We need to build on these hard-learned lessons from responding to the COVID-19 pandemic and rapidly apply them to improving the response to the AMR global health crisis. Now is the time to halt the continued spread of AMR by advancing novel and innovative strategies for: improved surveillance of drug-resistant pathogens; discovery, preclinical and clinical evaluation of safe and effective antimicrobials and other therapeutics; development and validation of diagnostics capable of rapidly identifying drug-resistant pathogens and differentiating between bacterial and viral infections; identifying and testing preventive interventions including safe and effective vaccines; investigating and developing strategies utilizing bacteriophage therapy; combining genomic sequencing with artificial intelligence/machine learning tools, as well as educating healthcare providers and the public on the urgent need to increase antibiotic stewardship.

The development of antimicrobials is an essential step in addressing the AMR crisis; however, from 2017 to 2022 only 12 new antibiotics have been approved by the US FDA and/or the EMA. The majority of these newly approved antibiotics demonstrate limited clinical benefit over existing treatments and approximately 80% of these drugs are from existing classes of antibiotics where mechanisms of resistance have previously been demonstrated.^6^ As of late 2021, there were 77 antibiotic or combination agents and 217 antibacterial candidates in the clinical and preclinical development pipelines, respectively, targeting the 13 WHO priority pathogens, including *Mycobacterium tuberculosis* and *Clostridioides difficile.*^6^ The limited number of potential antimicrobials in these pipelines are inadequate to counter the rapid spread of AMR, as well as the continual occurrence of drug resistance and broad-spectrum AMR demonstrated by many pathogens.^2^

During the past several years, numerous targeted initiatives have been launched to increase the number of antimicrobials and other strategies to counter AMR across different stages of the pipeline from basic biomedical research through to clinical trials and regulatory approval. These ongoing activities have included the Combating Antibiotic Resistant Bacteria Biopharmaceutical Accelerator (CARB-X, estimated at 800 million USD), the Antimicrobial Resistance Multi-Partner Trust Fund (AMR MPTF, estimated at 100 million USD), the Global Antibiotic Research & Development Partnership (GARDP, estimated at 100 million USD), the Replenishing and Enabling the Pipeline for Anti-Infective Resistance Impact Fund (estimated at 125 million USD) and the AMR Action Fund (AMR, estimated at 1.1 billion USD). Most recently, the AMR Action Fund has awarded funds to two companies with novel approaches utilizing bacteriophages or β-lactam/β-lactamase inhibitors.^7^ The NIH and the Biomedical Advanced Research and Development Authority (BARDA) AMR Diagnostic Challenge awarded 20 million USD to support innovative, accurate, rapid, point-of-need medical diagnostic tests to detect drug-resistant pathogens or differentiate between bacterial and viral infections.^8^ Additionally, public agencies (NIH, BARDA) and private foundations and organizations worldwide are supporting key biomedical research that provides the basis for identifying new targets for antimicrobial agents, repurposing existing drugs, vaccines and other countermeasures to combat AMR. The UK and Sweden initially launched pilot subscription-based models to ensure pharmaceutical manufacturers of guaranteed contracts irrespective of the actual number of antibiotics sold. Based on the pilot program in the UK, the NHS has recently implemented the ‘Netflix’ subscription-style payment programme for two antibiotics, including cefiderocol and ceftazidime with avibactam.^9^ In the US, two congressional bills have been recently drafted, including the Developing an Innovative Strategy for Antimicrobial Resistant Microorganisms (DISARM) Act and the Pioneering Antimicrobial Subscriptions to End Upsurging Resistance (PASTEUR) Act, to stimulate the Federal Government’s and industry’s development and use of novel antimicrobial agents.^10^ At this time, only the PASTEUR Act is continuing to progress toward authorization.^11^

All of these programmes are highly commendable; however, more must be done now to fill the discovery and development pipelines and ultimately, pharmacists’ shelves, with the crucial antimicrobials, including oral and paediatric formulations, that are urgently needed. Our collective fight against AMR requires a reinvigorated, rededicated, coordinated and collaborative dedicated effort to accelerate the development of innovative and improved strategies to prevent, treat and diagnose AMR. Additional public and private funds are essential to support: continued biomedical research to identify new lead compounds and drug targets; strategies to de-risk pharmaceutical and biotech companies to fill the diagnostics, drug and vaccine pipelines; legislative and governmental incentives to encourage development of new and improved antimicrobials; and education of healthcare providers and the public on the need for antibiotic stewardship. This Call to Action requires that all of us—members of the pharmaceutical, biotechnology and medical diagnostic industries, along with healthcare providers, researchers in academia and government agencies, regulators, lawmakers, private investors and philanthropy communities join together to end the AMR crisis before it becomes an even greater public health emergency than the COVID-19 pandemic. Only through strategic and collective activities, at both the individual and global population level, can we hope to halt the further spread of AMR. These efforts targeting AMR must be a crucial component of our comprehensive plans and policies for pandemic preparedness and response. FasterCures and the Milken Institute stand ready to work with our public and private partners to stop AMR and prevent the impending AMR public health pandemic.
